# A Model of Online Temporal-Spatial Integration for Immediacy and Overrule in Discourse Comprehension

**DOI:** 10.1162/nol_a_00026

**Published:** 2021-01-01

**Authors:** Takahisa Uchida, Nicolas Lair, Hiroshi Ishiguro, Peter Ford Dominey

**Affiliations:** Ishiguro Lab, Graduate School of Engineering Science, Osaka University, Osaka, Japan; INSERM UMR1093-CAPS, Université Bourgogne Franche-Comté, UFR des Sciences du Sport, Dijon, France; Robot Cognition Laboratory, Marey Institute, Dijon, France; Ishiguro Lab, Graduate School of Engineering Science, Osaka University, Osaka, Japan; INSERM UMR1093-CAPS, Université Bourgogne Franche-Comté, UFR des Sciences du Sport, Dijon, France; Robot Cognition Laboratory, Marey Institute, Dijon, France

**Keywords:** discourse context, word embedding, N400, reservoir computing, word2vec

## Abstract

During discourse comprehension, information from prior processing is integrated and appears to be immediately accessible. This was remarkably demonstrated by an N400 for “salted” and not “in love” in response to “The peanut was *salted*/*in love*.” Discourse overrule was induced by prior discourse featuring the peanut as an animate agent. Immediate discourse overrule requires a model that integrates information at two timescales. One is over the lifetime and includes event knowledge and word semantics. The second is over the discourse in an event context. We propose a model where both are accounted for by temporal-to-spatial integration of experience into distributed spatial representations, providing immediate access to experience accumulated over different timescales. For lexical semantics, this is modeled by a word embedding system trained by sequential exposure to the entire Wikipedia corpus. For discourse, this is modeled by a recurrent reservoir network trained to generate a discourse vector for input sequences of words. The N400 is modeled as the difference between the instantaneous discourse vector and the target word. We predict this model can account for semantic immediacy and discourse overrule. The model simulates lexical priming and discourse overrule in the “Peanut in love” discourse, and it demonstrates that an unexpected word elicits reduced N400 if it is generally related to the event described in prior discourse, and that this effect disappears when the discourse context is removed. This neurocomputational model is the first to simulate immediacy and overrule in discourse-modulated N400, and contributes to characterization of online integration processes in discourse.

## INTRODUCTION

An astonishing aspect of human language comprehension is that it brings to bear a vast variety of information sources to the ongoing interpretation of words in language, and it does so without temporal penalties for accessing this information. Typically, search takes time, particularly when using artificial arrays of stimuli (Treisman, 1982). However, under naturalistic conditions, attentional mechanisms are recruited and search time is significantly reduced (Peelen & Kastner, 2014). The comprehension system similarly appears to have immediate access to diverse sources of stored information. This “immediacy assumption” posits that during comprehension, an attempt is made to relate each content word to its referent as soon as possible (Just & Carpenter, 1980). The immediacy assumption was initially developed in the context of reading and the visual fixation of words during reading (Just & Carpenter, 1980; Thibadeau, Just, & Carpenter, 1982).

N400 responses to words during reading have provided new evidence in favor of the immediacy assumption (Hagoort & van Berkum, 2007; Nieuwland & van Berkum, 2006; van Berkum, Hagoort, & Brown, 1999). The N400 is a scalp-recorded negativity that appears around 400 ms after words that are semantically anomalous, such as “socks” in “He spread his warm bread with socks,” and is one of the most robust EEG effects in language processing (Kutas & Hillyard, 1980). Since its discovery, the N400 has been extensively investigated and remains a key indicator of semantic processing (Kutas & Federmeier, 2011).

The immediacy effect has been remarkably demonstrated by a reversal of the expected response to the sentence “The peanut was salted/in love” with an N400 for “salted” and not for “in love.” The unexpected effect of an N400 in response to “The peanut was salted” was induced by providing a prior discourse that featured the peanut as an animate agent who was dancing, singing, and so forth (Nieuwland & van Berkum, 2006). In this context, Hagoort and van Berkum (2007, p. 802) elaborated the immediacy assumption as “the idea that every source of information that constrains the interpretation of an utterance (syntax, prosody, word-level semantics, prior discourse, world knowledge, knowledge about the speaker, gestures, etc.) can in principle do so immediately.” In such cases where discourse produces a modification to otherwise expected responses, we can say that there has been a “discourse overrule.”

A related study by Metusalem et al. (2012) further examined discourse overrule where prior discourse could establish a context that would modulate N400 responses to words by making them more or less acceptable in the discourse context. Remarkably, they demonstrated that a contextually anomalous word elicits a reduced N400 if it is generally related to the described event, but that this effect disappears when the discourse context is removed. These findings demonstrate that during the course of incremental comprehension, comprehenders activate general knowledge about the described event, which can produce a discourse overrule of the local linguistic stream.

This immediacy seems remarkable in the context of discourse models that require multiple distinct steps, such as the construction-integration model of Kintsch (1988). This highly productive and influential model of comprehension is based on the construction of an initial representation of the discourse based on the input, which requires (a) generating the propositional representation based on a parse, (b) elaborating these elements based on world knowledge, (c) inferring additional propositions, and finally (d), the formation of a connectionist network representation of the results of this constructive process. Once this construction is achieved, a second phase of integration takes place over multiple cycles of the network to generate a coherent and consistent network representation. Despite the fact that Kintsch was well aware of the timing of different comprehension and inference processes as examined in Till, Mross, and Kintsch (1988), the multistep specification of the construction-integration model gives the impression that comprehension over these multiple steps will take time. It is in this context that the immediacy observations can be considered remarkable.

This poses the puzzle of how these diverse sources of information can physically be made immediately available and allows us to ask two questions: First, how is the content of prior discourse made immediately available during language comprehension? Inevitably, that discourse context is itself constructed from prior knowledge encoded in the meanings of words, and so we cannot consider discourse without also considering the world knowledge encoded in word meaning. This leads to the second question: How is prior knowledge of the world, encoded in words, made immediately available during comprehension?

This reveals that there are two timescales of prior information to account for. One timescale is over the lifetime, and concerns the meaning of words as they have been encountered in contexts over extended periods of time. The second and shorter timescale is over the discourse and concerns the aggregation of words in the context of an event. We propose that in both cases, a form of temporal-to-spatial integration takes place, where a temporal sequence of experience is integrated into a distributed spatial (neural) representation. It is this distributed spatial pattern that will provide the immediate access to experience accumulated over different timescales.

### The Current Study

Our present research addresses these questions via two innovative aspects. The first is to take into account the accumulation of prior knowledge over the lifetime, through the use of techniques developed in machine learning to create word embeddings, which represent world knowledge from large natural language corpora that can be encoded in feed-forward networks. The second is to approximate how this knowledge is integrated during discourse comprehension through the use of a temporal-spatial integration function that can be implemented in recurrent cortical networks.

The use of recurrent networks to integrate word context for explaining the N400 has been employed in two recent models (Brouwer, Crocker, Venhuizen, & Hoeks, 2017; Rabovsky, Hansen, & McClelland, 2018). In both cases the models are trained on sentence corpora generated from relatively small vocabularies (35–74 words) where the semantics of words are handcrafted based on prespecified semantic features. The training corpora are carefully controlled by the experimenters, which allows for an explanation of the N400 for words in test sentences based on their contrast with structure encoded in the training corpora. These models account for a variety of N400 responses and do not attempt to address how discourse meaning can influence the N400 beyond the local sentence context. We explore a complimentary approach, where knowledge imparted to the model originates from the structure inherent in a large vocabulary, 3.5 billion word corpus of human-generated discourse. Semantic and structural representations are extracted from this vast corpus to create a vector space that can be used to generate word embeddings (Mikolov, Sutskever, Chen, Corrado, & Dean, 2013; Yamada et al., 2020).

Word embeddings or distributed word representations are *n*-dimensional vector representations of words that are generated by extracting statistics on neighbor relations between words in large text training corpora generated from human sources. In one of the classic approaches to this problem, Landauer and Dumais (1997) assumed that the psychological similarity between words is determined by how these words co-occur in samples of language and that the language producer generates language in a way that preserves this orderly mapping between semantic relatedness and output distance. Their latent semantic analysis (LSA) calculates statistics on word occurrences in documents based on this distributional hypothesis that assumes that words that are close in meaning will occur in similar pieces of text. The method can then provide vector representations for words and documents, and generate estimates of similarity. The model has had wide success both in information retrieval (for which it was initially developed) and in explaining a variety of language performance and cultural phenomena, including the psychology of vocabulary learning.

Research in distributional models for word embeddings continues to flourish, particularly as large corpora and computing resources become increasingly available. A prominent recent method is word2vec (Mikolov et al., 2013). Whereas LSA is based on counting of co-occurrences, word2vec is a distributional word embedding neural network model that is based on the prediction of words based on the surrounding context. The word2vec algorithm has had great success in a variety of natural language processing domains, and also captures a remarkable level of human social psychology and changes over an extended time (Garg, Schiebinger, Jurafsky, & Zou, 2018). In a milestone experiment to establish the link between human brain activity during word comprehension and high dimensional feature representations extracted from large corpora (similar to word2vec), Mitchell et al. (2008) tested the assumption that brain activity observed when thinking about concrete nouns could be derived as a weighted linear sum of contributions from each of its semantic features. They established a direct, predictive relationship between the statistics of word co-occurrence in text and the neural activation associated with thinking about word meanings.

Recent research has successfully extended these representation properties from word to text levels (Le & Mikolov, 2014), and further research indicates that the resulting distributed vector representations of stories can be related to distributed neural activity in the human default mode network while people read these stories (Dehghani et al., 2017). This allows us to consider the possibility that aspects of human neurophysiology in discourse comprehension can be modeled using distributed vector representations developed in machine learning for natural language processing. Indeed, these results suggest that distributed representations derived from large corpora encode not only word semantics, but also knowledge about the structure of human events as required for understanding stories (Dehghani et al., 2017). At the same time, while such research examines how word embeddings learned from large corpora may contribute to the understanding of human discourse processing, in computational linguistics, experimental protocols and results from human psycholinguistics are being used to improve understanding of what these language models actually know about language (Ettinger, 2020).

This leaves open the question of how different formats of input to this integrative process, including knowledge from the ongoing discourse and real-world knowledge, is orchestrated in the nervous system. In other words, how can word meaning be continuously integrated into a discourse representation consistent with semantic immediacy and discourse reversal? A response to this question can be provided by recurrent networks that simulate primate cortex (Dominey, 1995; Enel, Procyk, Quilodran, & Dominey, 2016; Hinaut & Dominey, 2013; Rigotti et al., 2013). This reservoir family of recurrent networks (Lukosevicius & Jaeger, 2009) eschews learning in the recurrent connections (Pearlmutter, 1995) and instead uses pre-established connections that allow much richer high dimensional dynamics. These recurrent networks have the desired property of maintaining an ongoing history of the past inputs that is continuously and instantaneously updated with each new input. Reservoirs have been used to provide online word processing during sentence comprehension that can generate P600-like responses to unpredicted grammatical structure (Hinaut & Dominey, 2013). (The P600 is a late positive event-related potential [ERP] that can be evoked by grammatical structure violations.)

Here we extend this approach by using a reservoir network to continuously maintain and immediately update a representation of the ongoing discourse, thus providing a response to the question of how discourse information is continuously and immediately integrated. The input to this discourse reservoir is the sequence of distributed vector representations of words in a text, which represents real-world knowledge. Given a sequence of word embeddings as input, the network is trained to generate the average vector as output. Average vectors have been demonstrated to serve as a functional approximation of a discourse representation (Cer, Diab, Agirre, Lopez-Gazpio, & Specia, 2017; Lilleberg, Zhu, & Zhang, 2015). The trained discourse reservoir thus generates a simple and neurophysiologically valid temporal-spatial integration of the discourse. This representation is compared with the vector representation of the target word, and the difference predicts the amplitude of the resulting N400 response. Our objective is to test the hypothesis that this temporal-spatial integration of word embeddings in a discourse reservoir can account for aspects of the immediacy assumption and the discourse overrule of local semantics in human discourse processing, as described in Hagoort and van Berkum (2007) and Metusalem et al. (2012). This includes prediction of N400 amplitudes in studies where amplitudes varied with experimental conditions (Metusalem et al., 2012). For comparison we will evaluate performance in predicting N400 for the discourse reservoir and the simple average vector representations.

The proposed model may be useful in psycholinguistic understanding of discourse comprehension because it takes a neurophysiologically motivated stance on the nature of event representations derived from discourse comprehension. One might intuit that the result of comprehension is the elaboration of a situation model with explicit coding of events, relations, agents, and their roles. An alternative incarnation of the situation model could be a distributed representation that indeed encodes these elements, but in a distributed embedding. The current study takes a first step in examining whether such distributed representations can be used to understand human performance in well-characterized comprehension tasks.

## RESULTS AND METHODS

The experiments are implemented in python, and all code and data are openly available for reproducing these experiments and performing new experiments. The code and data are available at https://github.com/pfdominey/DiscourseOverrule. See RunningExperiments-log.txt to get started.

### Word Embeddings

Word embeddings for English in all three experiments were generated using Wikipedia2Vec (Yamada et al., 2020), which learns word embeddings based on the Wikipedia corpus, which includes over 3 billion words in approximately 5.5 million articles. Transformer-based models like BERT (Devlin, Chang, Lee, & Toutanova, 2019) encode sentence context and yield an impressive performance on linguistic tasks such as inference classification, question answering, and sentence continuation. These models can generate different embeddings for the same word, dependent on the surrounding sentence context. In our case, we wanted to compare the potentially ambiguous single embedding of the target word to discourse representations that are aggregations of embeddings for single words in the text, using an aggregation method for which we have a neurophysiologically motivated model (described below). For this reason, we chose Wikipedia2Vec to generate these embeddings.

Wikipedia2Vec learns embeddings by jointly optimizing three submodels. The first is the word-based skip-gram word2vec model of Mikolov et al. (2013). Word2vec takes as its input a large corpus of text and produces a vector space, typically of several hundred dimensions, with each unique word in the corpus being assigned a corresponding vector in the space. Word vectors are positioned in the vector space such that words that share common contexts in the corpus are located close to one another in the space (Mikolov et al., 2013). The second submodel in Wikipedia2Vec is a link-graph model that learns entity embeddings by predicting the neighboring entities of each entity in the Wikipedia’s link graph. The third submodel is an anchor-context model that aims to place similar words and entities close to one another in the vector space using hyperlinks and their neighboring words in Wikipedia.

We used a pretrained Wikipedia2Vec model that was trained on the entire Wikipedia pages for March 20, 2018 (over 3 billion words in approximately 5.5 million articles). It should be noted that this is an extraordinary volume of human knowledge, in the sense that it has been established by and is used by an extensive community as an encyclopedic reference. In her analysis of Wittgenstein’s view on meaning, Nelson (2009, p. 276) paraphrases: “there is a constellation of uses of the word that implies conventional rules for use. The meanings of a word then emerge from its uses by a community of users.” As a form of encyclopedia, Wikipedia is a form of record of the community’s collective knowledge. While Wikipedia is based on words, the actual articles that describe each word are well formed text, often explaining historical contexts, summarizing novel plots, and so forth. This knowledge is represented in the corpus via the juxtaposition of words in the different Wikipedia articles. The training in Wikipedia2Vec is a form of temporal-spatial transformation that encodes this knowledge, as the temporal sequence of the training corpus is used to train a neural network that learns word embeddings.

When given an input word, the trained Wikepdia2Vec model returns that word’s representation in the high (in our case 100) dimensional space. Similarity between words can be calculated as the cosine of the angle between their two vectors, with 1 being identical, and 0 being orthogonal or completely unrelated. Here we provide an example of similarity measures of word embeddings generated with Wikipedia2Vec for pairs of related and unrelated words from Chwilla, Brown, and Hagoort (1995, p. 285, Appendix, translated from the Dutch *ader-bloed* and *zweet-tekst*):

Cosine similarity: (Related word pair) vein blood = 0.540219

Cosine similarity: (Unrelated word pair) sweat text = 0.165510

Chwilla et al. (1995) observed that when subjects were presented with such word pairs in a lexical decision task, the presentation of the second word in the pair produced a larger N400 amplitude for the unrelated word pairs.

### Predicting N400 Responses

Given this measure of similarity we can specify a linking hypothesis between these cosine similarity measures and N400 amplitude (Brouwer et al., 2017). Intuitively, the N400 is linked to semantic similarity, such that as semantic similarity decreases, N400 increases. Following the reasoning developed by Brouwer, the difference between the current state of discourse context and the next target word corresponds to the N400. In the experiments described below, we used this linking hypothesis as proxy for N400 amplitude.

$$N400=1-\cos\left(\text{context},\text{target-word}\right)$$

The context can consist of a single word, that is, the first word of a word pair, or it can be a more extended discourse of multiple sentences, as we now describe.

### Discourse Representations

Given a tool for generating word embeddings, and a linking hypothesis for predicting N400 amplitude between words, we extend this to generating the same type of representations for word sequences or discourse, which can correspond to single or multiple sentence texts. Discourse representations can be generated based on aggregations of single word representations (Lilleberg et al., 2015). A simple but effective aggregation method is to take the vector average of the words in the discourse. This includes the semantics of all of the constituent words, without information provided by word order, referred to as “the bag-of-words approach,” which has been extensively applied in automatic text classification (Joachims, 1998; Joulin, Grave, Bojanowski, & Mikolov, 2017). In tests of semantic similarity between sentences, a discourse embedding baseline was used, which involved averaging word embeddings and then using cosine to compute pairwise sentence similarity scores over these average vectors (Cer et al., 2017). Such state-of-the-art baselines for obtaining sentence embeddings based on averaging perform well on the benchmark data, though improved performance was obtained by more exotic algorithms. A related averaging method has been employed by Ettinger, Feldman, Resnik, and Phillips (2016) to simulate N400 responses observed by Federmeier and Kutas (1999).

This justifies our choice to employ the average Wikipedia2Vec as a simple but reliable model of discourse embedding for our experiments. Our rationale is to adopt a minimalist discourse aggregation method that is known to be effective and that can realistically be implemented in the recurrent reservoir model of temporal-spatial integration.

### Reservoir Model of Discourse Embedding: The Discourse Reservoir

We employed reservoir computing as a neurophysiologically valid model for the formation of these discourse representations. Reservoir computing exploits the omnipresent feature of local recurrent connections in the primate cortex (Goldman-Rakic, 1987) in order to generate high dimensional representations of the input sequence. It was first developed in the context of simulating neural activity in the prefrontal cortex of primates that performed sensorimotor sequence learning tasks (Dominey, Arbib, & Joseph, 1995), then in more general neural computation (Maass, Natschlager, & Markram, 2002) and signal processing (Jaeger & Haas, 2004). More recently, reservoir computing has been confirmed as a model of primate cortical function in higher cognitive processes (Enel et al., 2016; Rigotti et al., 2013). The underlying concept that differs from other recurrent network models is that the recurrent connections are fixed, and learning occurs in a readout from the reservoir units. By avoiding simplifications of the recurrent dynamics required for learning (Pearlmutter, 1995), this allows the recurrent reservoir network to have extremely rich high dimensional states that can then be used to learn essentially universal functions of the input (Maass et al., 2002).

We trained the reservoir so that when provided with a sequence of word embeddings as input, it would generate the average vector as output. The reservoir experiments were performed with the easyesn toolkit (https://pypi.org/project/easyesn/), a well-documented and easy to use toolkit. All simulations used a standard echo state network with 100 reservoir units, and input and output dimensions of 100 (the same as the word and discourse embedding dimensions). The reservoir was trained on a subset of the Wikipedia corpus made up of 4,000 articles, which generated a set of 1.5 million words. The input was this sequence of words in their Wikipedia2Vec representation, and the output was the average vector sequence, that is, avg(w1), avg(w1, w2), avg(w1, w2, w3), … avg(w1 … wN). The reservoir was trained using standard parameters specified by easyesn: leakingRate=0.2, regressionParameters=[1e−2], solver="lsqr", feedback=False, with no feedback from readout to input. Training required less than two minutes on an Intel® Core™ i5-8250 CPU @1.6 GHz, with 16 Go RAM. The training error is on the order of 0.068 over multiple tests.

**Figure 1.**
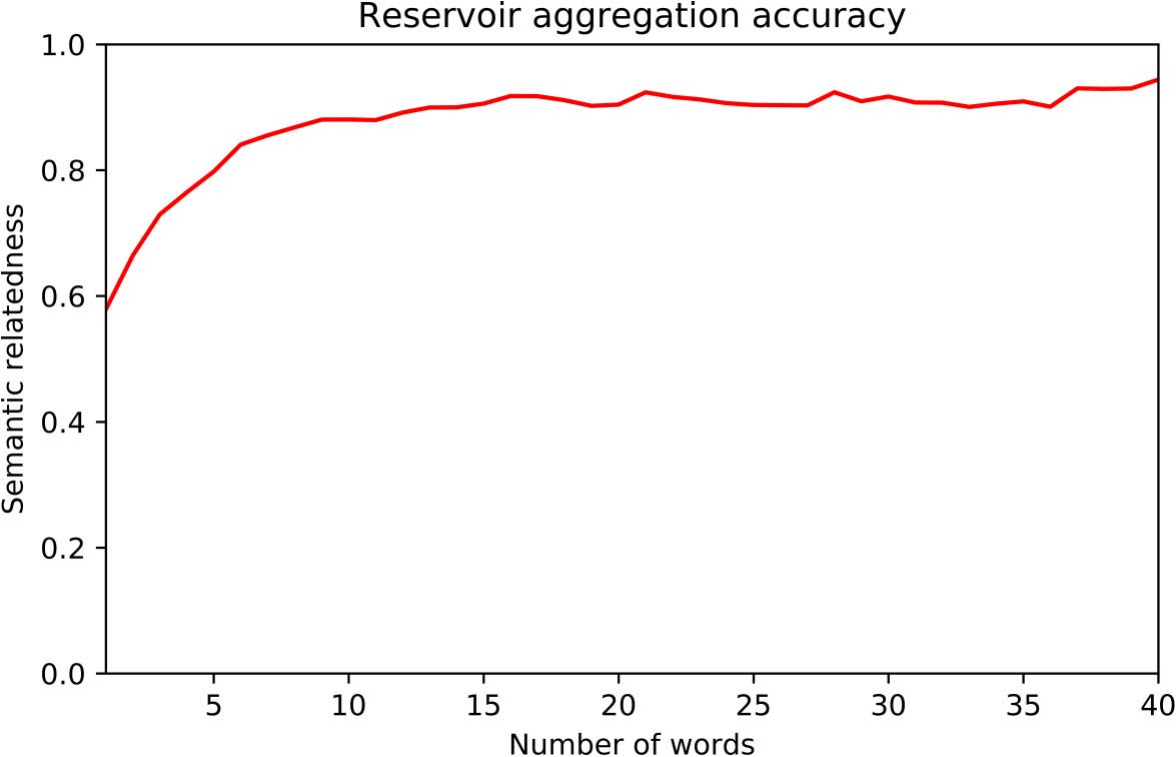
Cosine similarity (semantic relatedness) of actual average and reservoir generated average.

We evaluated the ability of the trained Discourse Reservoir to generate the average vector for increasing discourse lengths. The accuracy as measured by the cosine of the predicted and real average is presented in Figure 1. We found that after five words the similarity is over 0.8, and after 10 words over 0.9.

In the following experiments we used both methods of aggregation: Average-Embedding and Discourse Reservoir. In these experiments, we took a systematic approach, first validating the simple embedding method with an experiment examining semantic priming in word pairs, Chwilla et al. (1995), and then discourse effects on N400 with experiments from Nieuwland and van Berkum (2006), Hagoort and van Berkum (2007), and Metusalem et al. (2012).

### Experiment 1: N400 Semantic Priming Effect (Chwilla et al., 1995)

To validate that the semantics of Wikipedia2Vec word embeddings can be used to simulate human N400 responses, we first used our model to predict N400 responses in a classic task of semantic priming. One of the most robust findings in language psychology is semantic priming, where words are processed more efficiently when they are preceded by a semantically related word (Neely, 1991). Semantic priming has been revealed in ERP studies as the N400 semantic priming effect, where the N400 responses to words are increased when those words are preceded by a semantically unrelated word (Kutas & Van Petten, 1988). The objective of our first experiment was to test the hypothesis that Wikipedia2Vec encoding of semantic world knowledge can be used to predict human N400 responses to matching and nonmatching word pairs. In a lexical decision task, Chwilla et al. (1995) measured N400 responses to words that were distributed in pairs where the second word was either semantically related or not to the first word. Increased N400 response was reliably elicited for the unrelated pairs.

In Experiment 1, we thus used the stimuli from Chwilla et al. (1995), translated from Dutch to English, in order to validate the Wikipedia2Vec method for producing embeddings that can be used to measure semantic similarity. We generated the embeddings for the 40 related and 40 unrelated word pairs, and then used the cosine similarity, or semantic relatedness, between word pairs in the related and unrelated lists to predict N400s. Based on the linking hypothesis, the N400 amplitude is 1 − cosine similarity.

**Figure 2.**
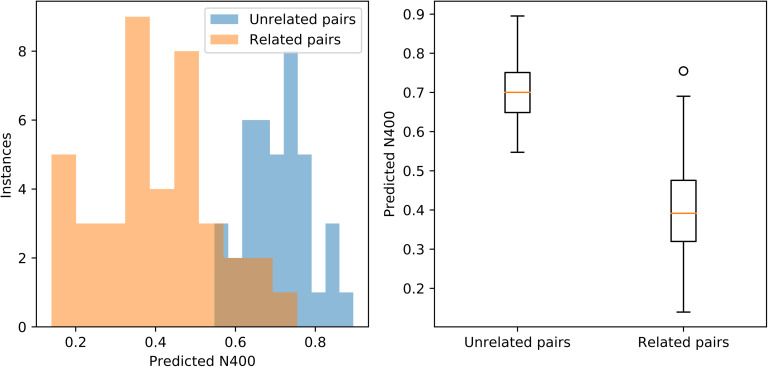
Distribution of predicted N400 for Related and Unrelated pairs. Related and Unrelated word pair list data from Chwilla et al. (1995). *Left panel:* Unrelated pair distribution shifted to right, with higher N400s. *Right panel:* Predicted N400 scores for related and unrelated pairs. Graphic notation: The box extends from the lower to upper quartile values of the data, with a line at the median. The whiskers extend from the box to show the range of the data. Circles indicate outliers.

Figure 2 illustrates the predicted N400 measures for related and unrelated pairs. The model predicts that related pairs have a smaller N400 (mean = 0.39) than unrelated pairs (mean = 0.70). This is highly significant, with *t*(39) = −11, *p* < 0.0001. The predicted N400 amplitude is significantly smaller for related versus unrelated pairs, just like the N400 amplitude found by Chwilla et al. (1995). This is consistent with the hypothesis that the Wikipedia2Vec embeddings can be used to predict human semantic similarity as revealed by the N400 response. It leaves open the question as to how this works, and whether the vector distance and the N400 are measuring the same process.

### Experiment 2: Accommodating the Peanut in Love (Nieuwland & van Berkum, 2006)

Nieuwland and van Berkum (2006) and Hagoort and van Berkum (2007) argued that discourse comprehension is realized by a single step model for integration of multiple knowledge sources, in which locally and globally supplied information can immediately effect semantic processing. They tested this model in ERP experiments with discourse set-up conditions where an inanimate object (e.g., a peanut) could take on the semantics of animacy (e.g., being in love). They argued that if discourse context prevails over animacy and real-world plausibility, then locally plausible but contextually inappropriate predicates (e.g., “the peanut was salted”) should elicit an N400 effect compared to locally anomalous but contextually appropriate predicates (e.g., “the peanut was in love”). One of their classic experimental texts that generates such locally plausible but contextually inappropriate predicates is presented in Table 1.

**Table 1.** A text that creates a locally plausible but contextually inappropriate context for the text in bold “The peanut was salted.”

The Peanut. A woman saw a dancing peanut who had a big smile on his face. The peanut was singing about a girl he had just met. And judging from the song the peanut was totally crazy about her. The woman thought it was really cute to see the peanut singing and dancing like that. **The *peanut* was salted/in love** and by the sound of it this was definitely mutual. He was seeing a little almond.

*Note*. Adapted from “When peanuts fall in love: N400 evidence for the power of discourse,“ by M. S. Nieuwland & J. J. van Berkum, 2006. *Journal of Cognitive Neuroscience*, *18*, p. 1106.

Using such discourses, Nieuwland and van Berkum (2006) and Hagoort and van Berkum (2007) were able to demonstrate a discourse overrule of expected N400 amplitudes where, indeed, a greater N400 was observed in response to the “The peanut was salted” versus “The peanut was in love.” In order to first determine whether according to Wikipedia2Vec “The peanut was salted” is more semantically acceptable than “The peanut was in love,” we first simply made the comparison of Wikipedia2Vec representations for “peanut” versus “love” and “peanut” versus “salted.” The 100 dimensional vector word embeddings were generated for peanut, love, and salted, using the trained Wikipedia2Vec model. Semantic relatedness for a pair of words was calculated as the cosine of their two respective vectors, and the predicted N400 was generated using the linking hypothesis (N400 = 1 − semantic relatedness) yielding the following results:

Predicted N400 "peanut" "love": 0.69

Predicted N400 "peanut" "salted": 0.34

Given this confirmation, we then set out to determine if the reversal of such effects could be predicted using our discourse embedding methods. We used the text in Table 1 (after removing stop-words like “a,” “had,” and “his,” and adding one neutral word at the beginning to prime the reservoir) as input to the discourse aggregation by average embedding and by the Discourse Reservoir. We truncated the text at the last occurrence of the word “peanut” (in bold italics in Table 1) so that the first and last words of the discourse are “peanut.” After each word in the discourse was presented, we calculated the Predicted N400 between the ongoing discourse aggregate and the words “love” and “salted.” Thus, first and last points of comparison are with the same discourse word “peanut” but without and with the discourse, respectively.

**Figure 3.**
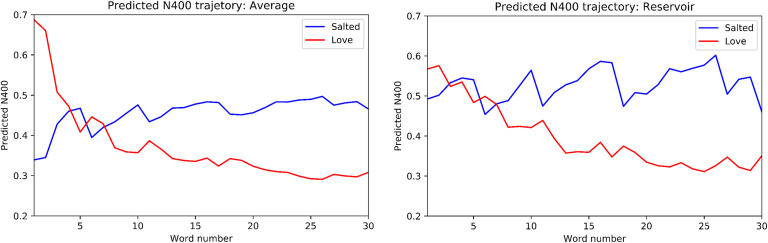
Trajectory of Predicted N400 values comparing the discourse aggregate vector with the vectors for “salted” (blue) and “love” (red), respectively. *Left panel:* Simple average aggregate. *Right panel:* Aggregate as calculated by the reservoir. Note the progressive shift from the canonical animacy and the real-world plausibility at the beginning of the discourse (where “salted” has a high proximity with the discourse vector and thus lower N400) to consistency with animacy of the peanut by the end of the discourse (where “love” has a higher proximity with the discourse vector and thus lower N400).

The results are presented in Figure 3, where we observe that the predicted N400 between the discourse aggregate and “salted” and “love” reverses during the elaboration of the discourse. At the outset the predicted N400 values are close to those listed above for the direct comparisons of “peanut” with “love” and “salted,” respectively. As successive words are added, the profile reverses. Figure 3 also displays results for the same experiment performed using the Discourse Reservoir. We observe that the differences for “salted” and “love” are smaller at the beginning of the discourse. This is because the reservoir does not generate a perfect average (which would be just a copy of the input for a discourse of one word) at the outset. Again we see the reversal of the predicted N400 over the elaboration of the discourse.

**Figure 4.**
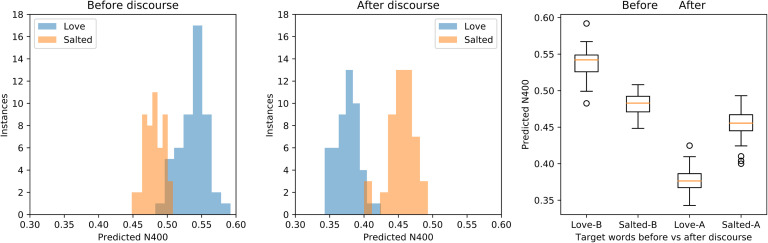
Predicted N400 scores for the online discourse embedding versus “love” and “salted” at the beginning and end of the Peanut discourse. *Left and middle panels:* Distribution histograms of scores for 50 Discourse Reservoirs at the beginning and end of the discourse, respectively. Note the N400 reversal with Love > Salted before, and Love < Salted after the discourse. *Right panel:* Box-whiskers plot with median, quartile, and range for “love” and “salted” before and after the discourse. The predicted N400 for the online discourse embedding and “peanut” versus “love” and “salted” are clearly reversed or overruled by the discourse.

In order to verify the generalization and robustness of this behavior, we performed the experiment with 50 distinct instances of the Discourse Reservoir, formed by using different initialization conditions for the connections in the reservoir. This corresponds to different subjects in a human experiment. Distributions of predicted N400 between the online discourse representation in the Discourse Reservoir and the vectors for “salted” and “love” at the outset of the discourse are shown in Figure 4, along with distributions after the discourse. We observe a clear and striking reversal of the predicted N400 between the online discourse representation and the vectors for “love” and “salted” before and after the discourse. We also observe that overall the predicted N400 scores are lower after the discourse.

These observations were confirmed with a repeated measures ANOVA on the predicted N400 values, with the factors Discourse (Beginning and End) and Target (Salted vs. Love). There was a significant main effect for Discourse, *F*(1, 49) = 1563, *p* < 0.001, with predicted N400 amplitude significantly smaller for End (0.416) than Beginning (0.510). There was a marginally significant effect for Target, *F*(1, 49) = 12, *p* < 0.005, with Love (0.457) less than Salted (0.468). Importantly, confirming the principal result, there was a significant Discourse × Ending interaction, *F*(1, 49) = 683, *p* < 0.0001, corresponding to the reversal of predicted N400 for Salted and Love between the Beginning and Ending conditions, as seen in Figure 4.

This demonstrates that online temporal-spatial integration of word embeddings in the Discourse Reservoir reproduces semantic immediacy and discourse overrule. In the simulation of the van Berkum Peanut experiments, the N400 response to “Peanut in love” was reversed by discourse providing context about animacy. The Discourse Reservoir network creates a discourse representation that models how human responses to words in discourse can be influenced by the preceding history of the discourse. By using an aggregation model that is updated online, the influences of the prior discourse are made immediately available. This provides a functional model of how semantic immediacy and discourse reversal can be obtained.

### Experiment 3: Event Knowledge Modulates Discourse Overrule (Metusalem et al., 2012)

Experiment 2 demonstrated how incrementally integrating word vectors into an online discourse vector provides an explanation for immediate discourse reversal. Experiment 3 examined this phenomenon in its component parts, strictly controlling the presence or absence of contextual discourse, to predict relative N400 amplitudes as discourse activates general knowledge about a described event, so that a contextually anomalous word elicits a reduced N400 if it is generally related to the described event, as observed by Metusalem et al. (2012). Metusalem et al. tested subjects on 72 scenarios that allowed manipulation of whether a discourse context would “rescue” the processing of a target word. An example of their scenarios is illustrated in Table 2.

**Table 2.** Example of one of the 72 discourse context scenarios

1. Elizabeth was standing at the intersection waiting for the light to change.
2. All of a sudden she saw a car barrel through the red light.
3. A moment later, she heard a terrible
4. (Expected) CRASH
5. (Unexpected-Related) POLICEMAN
6. (Unexpected-Unrelated) CONDUCTOR

*Note*. Adapted from “Generalized event knowledge activation during online sentence comprehension,” by R. Metusalem et al., 2012. *Journal of Memory and Language*, *66*, pp. 545–567.

In Metusalem et al.’s (2012) first experiment illustrating the effects of discourse context, subjects were exposed to a short discourse composed of sentences 1–3, with sentence 3 then ending in one of the three possible completions 4–6, that is, either Expected (4), Unexpected-Related (5), or Unexpected-Unrelated (6). N400 responses to the three completions revealed a clear effect of the context provided by sentences 1–2, as it “rescued” the N400 for the Unexpected-Related condition (POLICEMAN), which lay statistically between the small N400 for the Expected condition (CRASH) and the large N400 for the Unexpected-Unrelated condition (CONDUCTOR).N400 amplitude in the Unexpected-Unrelated condition was significantly greater (i.e., more negative) than the Unexpected-Related condition, which in turn was greater than the Expected condition. N400 amplitudes followed the significant progression: Unexpected-Unrelated > Unexpected-Related > Expected.

Given this demonstration of the effects of discourse context, Metusalem et al. (2012) then performed a second experiment using “No Context” to determine what would happen under the same conditions but in the absence of the context provided by the first two sentences of the scenario. In terms of the example in Table 2, subjects were exposed only to sentence 3—thus the target word 4 “CRASH” remains expected; the target words 5 “POLICEMAN” and 6 “CONDUCTOR” are both unexpected; and “POLICEMAN” loses its status of being related to the event. Metusalem et al. observed that the rescuing of the N400 for the Unexpected-Related disappeared, with the N400 profile of a large and equivalent N400 for both Unexpected cases with respect to the small N400 for the Expected case: Unexpected-Unrelated = Unexpected-Related > Expected.

In summary, when a discourse context was present, the N400 effect for an unexpected but semantically related word was rescued, reducing its N400 amplitude.

#### Simulation of 72 Metusalem et al. (2012) scenarios with the average vector

**Figure 5.**
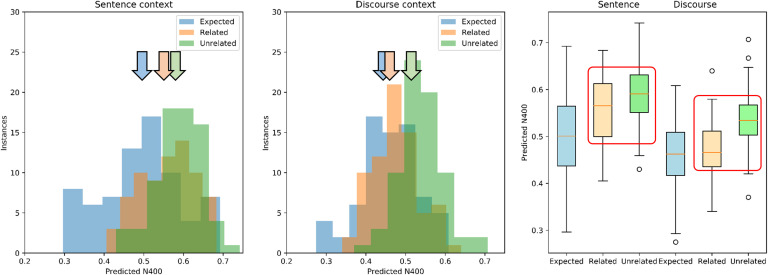
Predicted N400 for Expected, Related, and Unrelated words as a function of discourse context using Wikipedia2Vec average discourse aggregation. *Left and middle panels:* Distribution histogram of Predicted N400 scores for the three target word types as a function of the context—Sentence versus Discourse—using the Wikipedia2Vec average discourse aggregation. *Left panel:* Sentence context. Predicted N400 scores for Expected words are distributed to the left, with respect to Related and Unrelated, which are superimposed with similar increased values. *Middle panel:* Discourse context. The distributions for Related and Unrelated become separated with values for Related words shifting to the left, reflecting the decrease in their Predicted N400 induced by the discourse. Vertical colored arrows indicate means: Note the shift of Related to the left when passing from sentence to discourse. *Right panel:* Comparison of Predicted N400s between discourse vector and target words after the Sentence and after the complete discourse with context. With sentence only, the Expected word has a smaller Predicted N400 than the Unexpected-Related and Unexpected-Unrelated. With complete discourse, the N400 for the Unexpected-Related ending is rescued.

We set out to determine if such discourse rescue effects could be predicted based on our hypothesis that a temporal-spatial integration of word embeddings can account for aspects of immediacy and discourse overrule. We first tested discourse aggregation using the average vector in two conditions for each of the 72 scenarios. In the first, only sentence 3 was used as input to the vector average. We then calculated the predicted N400 as the 1 − cosine of the vectors representing the aggregated discourse and the target word for each of the three target words. This resulted in three predicted N400 measures, Sentence versus Expected, Sentence versus Unexpected-Related, and Sentence versus Unexpected-Unrelated. We then repeated this procedure, using sentences 1–3 as the input to generate the average vector. Again, we measured the predicted N400 given the discourse representation, in response to the three target words. This yielded six measures for each of the 72 scenarios. Expected, Unexpected-Related, and Unexpected-Unrelated with Sentence versus Discourse context. The results for these measures are illustrated in Figure 5.

The distributions of predicted N400 scores for the 72 scenarios are shown in Figure 5. In the Sentence context, predicted N400s for the Expected responses are distributed to the left, with lower values (see Figure 5, left panel). The impact of using the extended Discourse to generate the context is evident, as the scores for the Unexpected-Related words shift to the left, with smaller N400s (see Figure 5, middle panel). In the Sentence condition, a smaller predicted N400 is shown (Figure 5, right panel) for the Expected words and larger predicted N400 for the Unexpected-Related and Unexpected-Unrelated. In the Discourse condition, again a lower predicted N400 is shown (Figure 5, right panel) for Expected words, but now the predicted N400 for the Unexpected-Related words has diminished, and appears smaller than that for the Unexpected-Unrelated words. This rescue of the N400 by discourse is the effect of discourse overrule.

These observations were confirmed using 2 × 3 ANOVA on the Predicted N400 score, with factors Context (Sentence, Discourse) and Relatedness (Expected, Unrelated-Related, Unexpected-Unrelated). There was a significant main effect for Context, *F*(1, 71) = 138.3, *p* < 0.001, with the predicted N400s smaller for Discourse (0.491) than Sentence (0.547) conditions. There was a significant main effect for Relatedness, *F*(2, 71) = 36.7, *p* < 0.0001, with Expected (0.487) smaller than Unexpected-Related (0.517), smaller than Unexpected-Unrelated (0.563). Importantly there was a significant Context × Relatedness interaction, *F*(2, 142) = 14.8, *p* < 0.0001. This corresponds to the observation that for the Sentence condition, the predicted N400 for Expected (0.495) is significantly less than Unexpected-Related (0.559), which is significantly less than Unexpected-Unrelated (0.598), while in the Discourse condition, Expected (0.459) and Unexpected-Related (0.465) are not significantly different (*p* = 0.1), and both are less than Unexpected-Unrelated (0.598). Thus, the presence of the extended discourse reduces the predicted N400 for the Unexpected-Related, but not Unexpected-Unrelated words. This reflects the discourse overrule behavior observed in human subjects by Metusalem et al. (2012), where the Unexpected-Related is rescued by the extended discourse. This is consistent with their observation that generalized event knowledge provided by discourse contributes to mental representations of described events, is immediately available to influence language processing, and likely drives linguistic expectancy generation.

#### Simulation of 72 Metusalem et al. (2012) scenarios with the discourse reservoir

**Figure 6.**
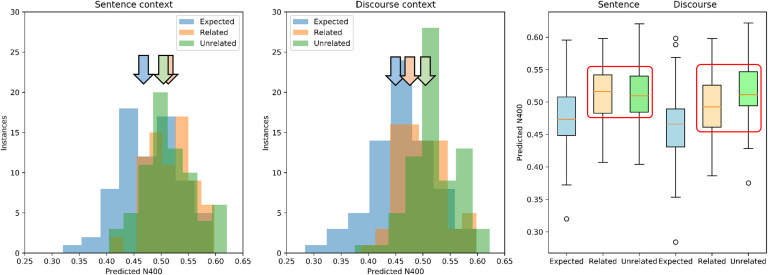
Predicted N400 as a function of discourse context using Wikipedia2Vec reservoir discourse aggregation. *Left and middle*: Distribution histogram of Predicted N400 scores for the three target word types as a function of the Context: Sentence versus Discourse. Vertical arrows indicate mean and allow visualization of shifts between sentence and discourse contexts. *Left panel:* Sentence context. Predicted N400 scores for Expected words are distributed to the left, with respect to Related and Unrelated, which are superimposed with similar reduced values. *Middle panel:* Discourse context. The distributions for Related and Unrelated become separated, with values for Related words shifting to the left, reflecting the decrease in their Predicted N400 induced by the discourse. *Right panel:* Predicted N400s for Discourse Reservoir vector and target words after the sentence and after the complete discourse with context. With sentence only, the Expected word has a smaller Predicted N400 than the Unexpected-Related and Unexpected-Unrelated. With complete discourse, the Predicted N400 for the Unexpected-Related ending is rescued.

In order to evaluate its discourse aggregation capability, we then tested a single instance of the Discourse Reservoir in two conditions for each of the 72 scenarios in Metusalem et al. (2012), following exactly the same procedure as for the vector average, but now using the reservoir. As in the previous experiment, in the first condition, only sentence 3 was used as input, this time to the Discourse Reservoir network that had been trained to generate the vector average of its inputs as described above. We then calculated the predicted N400 as 1 − cosine of the vectors representing the aggregated discourse and the target word for each of the three target words. This resulted in three predicted N400 measures: Sentence versus Expected, Sentence versus Unexpected-Related, and Sentence versus Unexpected-Unrelated. We then repeated this procedure, using sentences 1–3 as the input to the reservoir, which had been trained to generate the average vector. Again, we calculated the predicted N400 between the discourse representation and the three target words. This yielded six measures for each of the 72 scenarios: Expected, Unexpected-Related, and Unexpected-Unrelated with the Sentence and Discourse contexts. The results for these measures are illustrated in Figure 6.

The distributions of predicted N400 scores for the 72 scenarios are shown in Figure 6. In the Sentence context, these are comparable to the results of the vector average discourse aggregation results in Figure 5. N400 predictions for the Expected responses are distributed to the left, with lower values. The impact of using the extended Discourse to generate the context is evident (see Figure 6, middle panel), as the scores for the Unexpected-Related words shift to the left, with smaller N400s. For the Sentence condition, a smaller predicted N400 for the Expected words is shown (Figure 6, right panel), along with increased predicted N400 for the Unexpected-Related and Unexpected-Unrelated. In the Discourse condition, again is shown (Figure 6, right panel) a reduced predicted N400 for Expected words, but now the predicted N400 for the Unexpected-Related words has decreased, and appears smaller than that for the Unexpected-Unrelated words.

These observations were confirmed using 2 × 3 ANOVA on the predicted N400 score, with factors Context (Sentence, Discourse) and Relatedness (Expected, Unexpected-Related, Unexpected-Unrelated). There was a significant main effect for Context, *F*(1, 71) = 13.48, *p* < 0.001, with the predicted N400 smaller for Discourse (0.492) than Sentence (0.501) conditions. There was a significant main effect for Relatedness, *F*(2, 71) = 28.17, *p* < 0.0001, with N400s for Expected (0.468) smaller than Unexpected-Related (0.503), smaller than Unexpected-Unrelated (0.518). Importantly there was a significant Context × Relatedness interaction, *F*(2, 142) = 18.95, *p* < 0.0001. This corresponds to the observation that for the Sentence condition, N400s for Unexpected-Related (0.511) and Unexpected-Unrelated (0.515) are not significantly different (*p* = 0.72), while in the Discourse condition, they are significantly different with Unexpected-Related (0.496) significantly smaller than Unexpected-Unrelated (0.522) *p* < 0.0001. This decrease in the predicted N400 of the Unexpected-Related words reflects the discourse overrule behavior observed in human subjects by Metusalem et al. (2012).

#### Simulation of 72 Metusalem et al. (2012) scenarios with 50 discourse reservoirs

**Figure 7.**
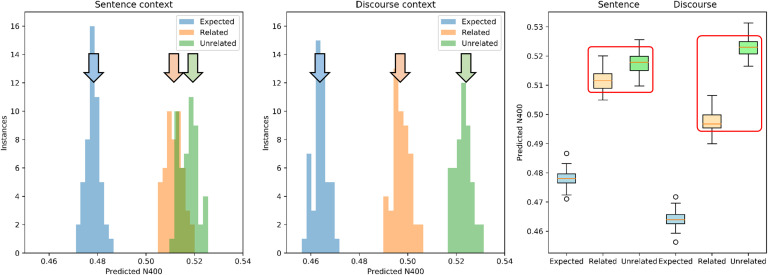
Distribution histogram of Predicted N400 scores for the three target word types as a function of the context—Sentence versus Discourse—for the 50 reservoirs, comparable to Figure 6 but with a more pronounced effect. *Left panel*: Sentence context. Predicted N400 scores for Expected words are distributed to the left, with respect to Related and Unrelated, which are superimposed with similar increased values. *Middle panel:* Discourse context. The distributions for Related and Unrelated become separated with values for Related words shifting to the left, reflecting the decrease in their Predicted N400s, rescued by the discourse. *Right panel*: Predicted N400s for Discourse Reservoir vector and target words after the sentence and after the complete discourse with context. With sentence only, the Expected word has a smaller Predicted N400 than the Unexpected-Related and Unexpected-Unrelated. With complete discourse, the Predicted N400 for the Unexpected-Related ending is rescued.

In order to evaluate the generalization and reliability of these results, we then performed the same experiment on 50 instances of the reservoir, corresponding to 50 experimental subjects. As in the previous case, each reservoir was tested with just the sentence and with the complete discourse for the Expected, Unexpected-Related, and Unexpected-Unrelated target words, for the 72 scenarios. The results are presented in Figure 7.

The distributions of mean predicted N400 values for the 50 subjects (i.e., instances of the reservoir) are shown in Figure 7. First, when compared with the results for a single reservoir in Figure 6, much less variability in the scores is found. This is similar to the variability across trials for a single subject (single reservoir) that is reduced when the means of those values are then averaged across multiple subjects (multiple reservoirs). This corresponds to the observation that for a single reservoir there is variability between scores in the different scenarios, and across multiple reservoirs there is less variability in the means averaged over the 72 scenarios for each reservoir. Importantly, a shift from right to left for the Unexpected-Related comparisons between Sentence to Discourse condition is shown (Figure 7, left and middle panels). In the Sentence condition we see a smaller predicted N400 for Expected words and increased predicted N400 for Unexpected-Related and Unexpected-Unrelated (see Figure 7, right panel). What is most remarkable is the effect of the extended Discourse in reducing the predicted N400 for the Unexpected-Related words, and increasing it for the Unexpected-Unrelated words.

These observations were confirmed using 2 × 3 ANOVA on the Predicted N400 score, with factors Context (Sentence, Discourse) and Relatedness (Expected, Unexpected-Related, and Unexpected-Unrelated). There was a significant main effect for Context, *F*(1, 49) = 3307, *p* < 0.001, with the predicted N400 smaller for Discourse (0.495) than Sentence (0.502) conditions. There was a significant main effect for Relatedness, *F*(2, 49) = 12490, *p* < 0.0001, with Predicted N400s for Expected (0.471) less than Unexpected-Related (0.505), less than Unexpected-Unrelated (0.520). Importantly there was a significant Context × Relatedness interaction, *F*(2, 98) = 3600, *p* < 0.0001. All post hoc comparisons were highly significant. The two Unexpected endings vary significantly from the Expected baseline in both Sentence and Discourse contexts. For the Sentence condition, Unexpected-Related (0.512) and Unexpected-Unrelated (0.518) significantly differ only by 0.006, while in the Discourse condition the significant difference between Unexpected-Related (0.497) and Unexpected-Unrelated (0.523) is greater at 0.026. A paired *t* test between these differences in Sentence versus Discourse is highly significant (*t* = 71, *p* < 10^−10^). In the presence of Discourse, the predicted N400 for Unexpected-Related decreases and Unexpected-Unrelated increases. Again, this decrease in the predicted N400 of the Unexpected-Related words in the Discourse condition reflects discourse rescue or overrule behavior observed in human subjects by Metusalem et al. (2012).

In summary, the principal result is that the model predicts that in the Discourse condition, the N400 for Unexpected-Related ending words is rescued from its high value in the Sentence condition, while this is not the case for the Unexpected-Unrelated endings. This corresponds to the principal result of the two experiments in Metusalem et al. (2012).

## DISCUSSION

This research was motivated to answer questions about how multiple sources of knowledge can be accessed during discourse processing in a way that respects the immediacy assumption and discourse overrule: How can world knowledge from lexical semantics and the ongoing knowledge from the accumulated discourse be immediately applied to the processing of the next word in the discourse to overrule expectations based on local semantics? A striking experimental observation that motivates such a question is the N400 response to the word “salted” in “The peanut was salted” when that sentence was preceded by a discourse context where the peanut was framed as an animate agent who was singing and dancing with happiness (Hagoort & van Berkum, 2007; Nieuwland & van Berkum, 2006). These experiments illustrated the effects of semantic immediacy and discourse overrule, where the local semantics are overruled by those of the preceding discourse in the immediate time frame of the N400.

We determined that satisfying the immediacy assumption and discourse overrule requires two coordinated capabilities corresponding to lexical retrieval and online discourse processing. In both cases if the data were processed in a purely serial manner, then the size of the dataset would tend to increase processing time. The larger the lexicon, the longer the processing. Similarly, as words accumulate in a discourse, serial retrieval would require time proportional to the length of the discourse to access information (such as the animacy of the peanut) that appeared earlier in the discourse. Yet in both cases, there seems to be no time penalty in human language processing. We believe that this is because the brain has adopted solutions that transform serial representations into distributed spatial representations. Sequential information is recoded in a distributed spatial form which can be immediately accessed by distributed neural connections.

For lexical retrieval and word semantics, this serial-to-spatial transformation is exploited in distributed vector representations that have been developed in the domain of natural language processing, which capture rich linguistic and conceptual information (Bengio, Schwenk, Senécal, Morin, & Gauvain, 2006; Landauer & Dumais, 1997; Mikolov et al., 2013). These systems can be implemented as statistical models or neural networks. The point is that they are corpus-based learning algorithms that generate distributed representations of word meanings and, thus, transform the temporal and sequential information in the training corpora into distributed spatial vector spaces that allow immediate access.

In Experiment 1, we illustrated how a current leading incarnation of these systems, Wikipedia2Vec, which is based on word2vec, displays human-like predictions of N400 responses for word pairs from a classic study of semantic priming and N400 (Chwilla et al., 1995). Clearly we do not want to claim that Wikipedia2Vec or other word embedding algorithms understand word meanings in all the ways that humans do. These methods do, however, capture a certain degree of the knowledge contained in the human-produced corpora on which they are trained, in the vector embeddings they generate. Characterizing the limits of these models remains an open topic of research (Ettinger, 2020). The geometric relationships between these vectors capture meaningful semantic relationships between the corresponding words in a variety of domains from analogical reasoning and developmental psychology to neuroscience (Dehghani et al., 2017; Landauer & Dumais, 1997; Mikolov et al., 2013; Mitchell et al., 2008). With respect to the neural correlates, studies of human brain imaging indicate that human brain activity during language comprehension can be represented and decoded using models based on distributed word representations from natural language processing in machine learning (Huth, de Heer, Griffiths, Theunissen, & Gallant, 2016). This suggests that understanding the highly distributed nature of the human semantic system may be aided by understanding the representational structure of high dimensional word and discourse embeddings. Indeed, these representations can be used to predict the meaning of brain activation at the level of words (Mitchell et al., 2008) and stories (Dehghani et al., 2017). It has even been shown that these methods can capture societal shifts reflected in language over decades, for example, the women’s movement in the 1960s and Asian immigration to the United States (Garg et al., 2018).

For integrating these word representations during discourse processing, we considered an approach where the temporal-sequential information in the discourse is transformed into a spatial representation via recurrent connections in a reservoir network. Previous research demonstrated how a reservoir model for learning grammatical constructions could take word sequences as input and generate a distributed spatial representation that was used to generate immediate responses based on the grammatical structure in the input, including simulation of the P600 ERP for structure violations (Hinaut & Dominey, 2013). It is now accepted that these recurrent reservoir networks model the functional neurophysiology of local recurrent connections in primate cortex in a variety of higher cognitive functions (Enel et al., 2016; Fusi, Miller, & Rigotti, 2016; Rigotti et al., 2013). In Experiments 2 and 3 we used the Discourse Reservoir to integrate word embeddings in a discourse, and we observed that this provided immediate access to accumulated information from the discourse, consistent with observations of human N400 responses (Hagoort & van Berkum, 2007; Metusalem et al., 2012; Nieuwland & van Berkum, 2006). Our results from Experiments 2 and 3 demonstrated how prior discourse could influence the processing of the current lexical item, shifting and even reversing predicted N400 responses based on prior discourse.

Again, we do not claim that the Discourse Reservoir (nor an average vector of the words in the discourse) models full human discourse comprehension. However, such simple discourse embeddings, which involve averaging word embeddings and then using cosine to compute pairwise sentence similarity scores, perform well and are used as baselines for evaluation of state-of-the-art algorithms for Semantic Textual Similarity evaluation tasks (Cer et al., 2017) and also have been used for modeling N400 in sentence contexts (Ettinger et al., 2016). Similarly, the use of averaging Wikipedia2Vec embeddings to characterize sentences was employed in the winning system at the Human–Computer Question Answering Competition at the Thirty-first Annual Conference on Neural Information Processing Systems (Yamada, Tamaki, Shindo, & Takefuji, 2018). Likewise, reservoir models have been demonstrated to be capable of integrating multiple words in short discourse for subsequent thematic role assignment (Hinaut & Dominey, 2013). Tasks that require access to discourse history, such as semantic role labeling, where the role of a current noun requires access to the verb and grammatical words that may be far behind in the past can benefit from this history preserving capability of recurrent networks (Zhou & Xu, 2015).

### Neural Implementation of the Model

The integration of discourse and world knowledge, and their interaction in terms of overall coherence in comprehension, has been shown to recruit distinct neural substrates (Menenti, Petersson, Scheeringa, & Hagoort, 2009). The left inferior frontal gyrus (IFG) was associated with processing world knowledge, and the right IFG was more sensitive to the effects of a “rescuing” in local discourse similar to the rescuing in discourse in the Metusalem et al. (2012) task. Interestingly, the left angular gyrus showed a significant interaction between world knowledge and discourse context, suggesting a role in tracking the coherence between the discourse context and the current sentence (Menenti et al., 2009).

The immediacy of these world knowledge and local context effects was characterized by Hald, Steenbeek-Planting, and Hagoort (2007) using EEG. They observed that the effects of manipulating world knowledge and discourse context modulated N400 responses in the same 325–525 ms time frame, illustrating the immediacy of these effects. The interplay between these two levels of lexical and discourse processing has been further revealed in recent studies of human electrophysiology during reading. Investigating the underlying mechanisms with MEG, Hultén, Schoffelen, Uddén, Lam, and Hagoort (2019) showed that within 500 ms of seeing a word, the word’s lexical information has been retrieved and unified with the sentence context. This happens via co-modulation between the left posterior temporal cortex and the left inferior frontal cortex for individual words around 400 ms after their onset across the sentence progression. The results provide a detailed description of the temporal orchestration related to single word processing in the context of ongoing language.

In terms of the spatial organization of these representations, Dehghani et al. (2017) calculated the distributed representations of stories and demonstrated that by using these representations, they could identify the specific story that a participant was reading from the neural data, based on a collection of brain regions most prominently located in the default mode network. That is, they established a predictive relation between high dimensional distributed story representations generated by doc2vec and high dimensional distributed story representations in the human default mode network. Once trained, their classifier could predict the doc2vec representation of a story, given the human subject’s brain activation from reading that story. This argues that, in addition to providing powerful tools for natural language processing, these distributed representations can help us to better understand biological mechanisms for discourse comprehension.

This allows the elaboration of a model of narrative processing where the left IFG is associated with processing lexical semantics and world knowledge, corresponding to our temporal-spatial transformation realized by the Wikipedia2Vec word embedding model. In this model, the right IFG is associated with the temporal-spatial integration processing of local discourse that contributes to the rescuing effects observed by Metusalem et al. (2012) and implemented as a recurrent reservoir network in our model. Such a model should also include observation of default mode network activity during narrative processing (Metusalem et al., 2012), which may correspond to embodied simulation of the represented events (Jouen et al., 2015).

### Related Neurocomputational Models

Our present study can be situated in the context of existing models that simulate the N400. Rabovsky et al. (2018) developed a semantic gestalt (SG) model that explains N400 results from a variety of human experimental studies. In their model, the incoming word is processed through a hidden layer where it combines with the previous SG activation, thus implementing a form of recurrent network. The model has an input vocabulary of 74 words that are associated with handcrafted semantic representations. Using this vocabulary, the model is trained on [sentence, event] pairs, where events are characterized as sets of role-filler pairs (e.g., agent, man; action, eat; patient, eggs; location, kitchen; situation, breakfast). The N400 response is modeled as the semantic update (SU) or sum of changes in activation in the SG network in response to the current word. The authors note that the SU at any given point is determined by the statistical regularities in the training corpus, and they explain how the observed effects depend on the training corpus. This research provides a detailed analysis of how the N400 arises as a function of how the current sentence is related to the probabilistic structure of sentences that the system has been previously exposed to. The SG representation itself clearly embodies the notion of temporal-spatial transformation that we advocate, and it has the further advantage that it can be used to generate an event representation of the meaning of the sentence. It will be interesting in the future to extend this model to address how discourse meaning can influence the N400 over and above the local sentence context (Rabovsky et al., 2018) as in the experiments of Nieuwland and van Berkum (2006) and Metusalem et al. (2012).

Brouwer et al. (2017) developed a model that addresses the N400 in the larger context of the N400 and P600. They develop a theory and model where the N400 reflects the retrieval of word meaning from semantic memory, and the P600 component indexes the integration of this meaning into the unfolding utterance interpretation. The word retrieval is contingent on the context of the utterance, which is accumulated by recurrent connections in the integration network. This accumulation allows for the immediacy effect. The results of the retrieval then feed forward into the integration network where the utterance level representation is developed, and where the integration effects corresponding to the P600 are simulated. Both the retrieval (N400) and the integration (P600) effects displayed by the model result from a confrontation at test time with the statistics that were accumulated by the model from the training corpus during training time. It will be interesting to determine how this model will address discourse effects as in the experiments of Nieuwland and van Berkum (2006) and Metusalem et al. (2012).

The above two models and the model we have developed here all share the use of recurrent networks to perform the temporal-spatial integration that provides for semantic immediacy. The models of Rabovsky et al. (2018) and Brouwer et al. (2017) rely on regularities that are introduced by the construction of the training corpus. Their models learn meaning representations and thus require labeled corpora of [sentence, meaning] pairs that must be created by the experimenters. Our research exploits regularities that are encoded in the distributional properties of a large, natural, human-generated corpus. We do not, however, include an explicit representation of the meaning. Future research should consider how these modeling approaches can be combined, for example, by bootstrapping a system using labeled training data to account for event coded meaning representations, and then extending this using large unlabeled data encoded in word embeddings.

The observation that we can simulate N400 effects, including multiple sentence discourse effects related to human event semantics, indicates that a trace of these regularities is represented in the information extracted by the Wikipedia2Vec algorithm. This is supported by studies from Ettinger (Ettinger, 2020; Ettinger et al., 2016) that confront state of the art language models, including word2vec (Mikolov et al., 2013) and BERT (Devlin et al., 2019), with psycholinguistics to determine what information the models are able to use when assigning probabilities to words in context. This research demonstrated how word2vec averages (Ettinger et al., 2016) and BERT (Ettinger, 2020) were able to account for data from several psycholinguistic experiments, including Federmeier and Kutas (1999). Federmeier and Kutas (1999) used stimuli similar to those of Metusalem et al. (2012). Context was established in a first sentence, followed by a second neutral sentence with final words that were either expected exemplars, within-category violations, or between-category violations, as in the example, *“Checkmate,” Rosaline announced with glee. She was getting to be really good at chess/monopoly/football*. Like Metusalem et al. (2012), they showed that N400s were present for the violations, but reduced for within category violations, particularly when the context was highly constrained. Ettinger et al. (2016) demonstrated this effect using word2vec averages to model the discourse context, and Ettinger (2020) demonstrated a preference for the expected versus violation endings using BERT. As Federmeier and Kutas (1999) did not perform a second experiment (as in Metusalem), they did not address the discourse induced effects in the presence and absence of the context sentence, but it is likely that they would have found similar effects, as we did for the Metusalem et al. (2012) experiments.

Future research should continue to exploit this fruitful interaction between computational linguistics and psycholinguistics. Fine characterization of language processing via psycholinguistics can continue to be used to better understand what computational language models are capable of (Ettinger, 2020), and computational models can continue to be used to characterize what the brain is doing (Dehghani et al., 2017; Huth et al., 2016; Huth, Nishimoto, Vu, & Gallant, 2012; Mitchell et al., 2008). A new domain of scientific discovery is emerging where these two approaches can actively cooperate.

In conclusion, we have presented results confirming our hypothesis that a temporal-spatial integration of word embeddings in a Discourse Reservoir can account for aspects of the immediacy assumption and discourse reversal in human discourse processing. This research offers several future perspectives. One is related to the limits of immediacy. Inference takes time (Calvo, 2000; Estevez & Calvo, 2000; Till et al., 1988), and it will be interesting to explore in more depth to what extent inferred knowledge is present in these systems, and how more extended inference mechanisms might be incorporated. Likewise, while we have focused on more immediate N400 effects, manipulations of discourse predictability can generate much later positivities (Brothers, Wlotko, Warnke, & Kuperberg, 2020; Brouwer, Fitz, & Hoeks, 2012), which reveal processes that should be accounted for in models of discourse processing (Brouwer et al., 2017; Brouwer et al., 2012).

One might consider that the type of modeling presented here does not help us understand human language, but rather that it helps us understand whether or not and how a computational model behaves like a human. Interestingly we can also proceed in the other direction. Our model defines a class of computations based on its two computational components, the language model that generates word embeddings, and the reservoir that accumulates discourse representations. The ability of the model to predict N400s during discourse comprehension allows us to hypothesize that some aspect of human neurophysiology is isomorphic to the model. This allows specific predictions to be made. One of the properties of the computational and cortical reservoir is the presence of mixed selectivity, that is, a nonlinear mixture of different task parameters in reservoir units that has been observed in the primate cortex and in reservoir models (Enel et al., 2016; Rigotti et al., 2013). We should thus expect to see evidence for mixed selectivity in human cortical electrophysiology during discourse comprehension, as potentially observable in MEG (Schoffelen et al., 2019).

Our research contributes to a more general principal of temporal-spatial transformation for cognitive immediacy. Standard conceptions of computation have been constrained by the Turing machine model and the Von Neumann architecture, which are inherently sequential in nature (Graves, Wayne, & Danihelka, 2014; Hopcroft, Motwani, & Ullman, 2001). If temporally structured sequences can be projected into a two dimensional space, and if computations can be realized in one step on representations in that space, then computational complexity is significantly reduced. Interestingly, this is exactly the organization of spatial maps in the brain, with extensive parallelism in the duplicated organization of the cortico-striato-thalamo-cortical circuits (Alexander, DeLong, & Strick, 1986). The further exploration of inherently spatial distributed computation will likely play a role in understanding human cognition and developing increasingly powerful learning systems.

## ACKNOWLEDGMENTS

Research supported by Region Bourgogne Franche-Comté (AAP 2019 RobotSelf), and the French Convention CIFRE N 2018/0434 Cloud-Temple/INSERM.

## FUNDING INFORMATION

Peter Ford Dominey, Conseil Régional de Franche-Comté (FR), Award ID: (AAP 2019 RobotSelf).

## AUTHOR CONTRIBUTIONS

Takahisa Uchida: Conceptualization, Investigation, Methodology, Software, Writing – Review & editing. Nicolas Lair: Conceptualization, Investigation, Methodology, Software, Writing – Review & editing. Hiroshi Ishiguro: Project administration, Supervision, Validation, Writing – Review & editing. Peter Ford Dominey: Conceptualization, Investigation, Methodology, Software, Supervision, Writing – Original draft.
